# Quantitative single-cell characterization of CAR+ T cell effector functions

**DOI:** 10.1186/2051-1426-1-S1-P36

**Published:** 2013-11-07

**Authors:** Ivan Liadi, Harjeet Singh, Gabrielle Romain, Nicolas Rey-Villamizar, Amin Merouane, Badrinath Roysam, Laurence J Cooper, Navin Varadarajan

**Affiliations:** 1 Chemical & Biomolecular Engineering, University of Houston, Houston, TX, USA; 2 Dept of Electrical Engineering, University of Houston, Houston, TX, USA; 3 Pediatrics, University of Texas M.D. Anderson Cancer Center, Houston, TX, USA

## 

Adoptive cell therapy (ACT) utilizing chimeric antigen receptor (CAR) T cells rendered specific for CD19 have demonstrated significant anti-tumor effects in patients with CD19+ chronic lymphocytic leukemia (CLL). In spite of the clinical promise of ACT in achieving complete responses, their efficacy remains unpredictable and new approaches are needed to address a priori define the therapeutic potential of T-cell based therapies. In our current work, we characterize the in vitro functionality of CD19-specific (CD19RCD28) CAR+ T cells propagated using artificial antigen presenting cells expressing membrane bound IL-21, by employing a novel methodology single-cell nanowell screening that determines their cytotoxic ability and cytokine secretion capability at single-cell resolution. We show that CAR+ T cells exert specific cytotoxicity against NALM6 cells (31 ± 8 %) when co-incubated at a 1:1 ratio in nanowell containers. Furthermore, single CAR+ T cells were capable of engaging and killing multiple targets; 17 ± 8% of T cells killed two target cells and 9 ± 3% killed three target cells within the 6 hour window of observation. In parallel, microengraving was used to determine the cytokine secretion profile of these same cells. Hierarchical clustering of the two functions indicated that interferon-gamma (IFNγ) secretion is not correlated to cytotoxicity or the ability of T cells to kill multiple target cells. Simultaneously, monitoring apoptosis on CAR+ T cells allowed us to quantify their activation-induced cell death (AICD). CAR+ T cells that secreted IFNγ upon target ligation did not undergo AICD whereas T cells that engaged in repeated killing showed an increased propensity to undergo AICD (p = 0.04). Dynamic time-lapse imaging of the interactions between CAR+ T cells and tumor cells indicated that the majority of CAR+ T cells have high basal motility, form long-lived interactions with tumor cells (50 - 100 min) that lead to motility arrest and subsequent tumor-cell apoptosis. However, contact lifetimes or overall contact duration were not reliable predictors of subsequent tumor-cell apoptosis. Finally, kinetics of serial killing suggest that motile CAR+ T cells that form short-lived contacts exhibit rapid killing with very little motility arrest in vitro. In summary, our SNS based methodology allows the deep functional characterization of clinical grade CAR+ T cells and can be used to: (1) determine in vitro functions of CAR+ T cells that correlate with clinical efficacy and (2) inform CAR design to maximize effector functionality while minimizing AICD.

